# Transcriptome analysis using next generation sequencing reveals molecular signatures of diabetic retinopathy and efficacy of candidate drugs

**Published:** 2012-05-02

**Authors:** Raj P. Kandpal, Harsha K. Rajasimha, Matthew J. Brooks, Jacob Nellissery, Jun Wan, Jiang Qian, Timothy S. Kern, Anand Swaroop

**Affiliations:** 1 Neurobiology-Neurodegeneration & Repair Laboratory, National Eye Institute, National Institutes of Health, Bethesda, MD; 2 Department of Basic Medical Sciences and Western Diabetes Institute, Western University of Health Sciences, Pomona, CA; 3 Department of Ophthalmology, Johns Hopkins School of Medicine, Baltimore, MD; 4 Department of Medicine, Case Western Reserve University School of Medicine, and Stokes Veterans Administration Hospital, Cleveland, OH; 5 Department of Pharmacology, Case Western Reserve University School of Medicine, and Stokes Veterans Administration Hospital, Cleveland, OH; 6 Department of Ophthalmology, Case Western Reserve University School of Medicine, and Stokes Veterans Administration Hospital, Cleveland, OH

## Abstract

**Purpose:**

To define gene expression changes associated with diabetic retinopathy in a mouse model using next generation sequencing, and to utilize transcriptome signatures to assess molecular pathways by which pharmacological agents inhibit diabetic retinopathy.

**Methods:**

We applied a high throughput RNA sequencing (RNA-seq) strategy using Illumina GAIIx to characterize the entire retinal transcriptome from nondiabetic and from streptozotocin-treated mice 32 weeks after induction of diabetes. Some of the diabetic mice were treated with inhibitors of receptor for advanced glycation endproducts (RAGE) and p38 mitogen activated protein (MAP) kinase, which have previously been shown to inhibit diabetic retinopathy in rodent models. The transcripts and alternatively spliced variants were determined in all experimental groups.

**Results:**

Next generation sequencing-based RNA-seq profiles provided comprehensive signatures of transcripts that are altered in early stages of diabetic retinopathy. These transcripts encoded proteins involved in distinct yet physiologically relevant disease-associated pathways such as inflammation, microvasculature formation, apoptosis, glucose metabolism, Wnt signaling, xenobiotic metabolism, and photoreceptor biology. Significant upregulation of crystallin transcripts was observed in diabetic animals, and the diabetes-induced upregulation of these transcripts was inhibited in diabetic animals treated with inhibitors of either RAGE or p38 MAP kinase. These two therapies also showed dissimilar regulation of some subsets of transcripts that included alternatively spliced versions of arrestin, neutral sphingomyelinase activation associated factor (Nsmaf), SH3-domain GRB2-like interacting protein 1 (Sgip1), and axin.

**Conclusions:**

Diabetes alters many transcripts in the retina, and two therapies that inhibit the vascular pathology similarly inhibit a portion of these changes, pointing to possible molecular mechanisms for their beneficial effects. These therapies also changed the abundance of various alternatively spliced versions of signaling transcripts, suggesting a possible role of alternative splicing in disease etiology. Our studies clearly demonstrate RNA-seq as a comprehensive strategy for identifying disease-specific transcripts, and for determining comparative profiles of molecular changes mediated by candidate drugs.

## Introduction

Diabetes has emerged as a major worldwide public health concern, and the number of diabetics is estimated to exceed 400 million by the year 2030 [1]. A side effect of diabetes, namely diabetic retinopathy, is also a leading cause of blindness in working age adults (NIH MedlinePlus the Magazine). Several approaches, including good glycemic control, use of blood pressure medications, and lipid control, have been demonstrated to inhibit diabetic retinopathy in clinical trials, but many patients are not able to maintain these regimens over the long-term. Thus, additional therapeutic approaches are continuously being sought. Several experimental therapies that include vitamin E, aspirin, aminoguanidine, or inhibitors of receptor for advanced glycation endproducts (RAGE) and p38 mitogen activated protein (MAP) kinase [2–6] have shown positive effects at inhibiting the development of diabetic retinopathy lesions in laboratory animals, but the underlying molecular mechanisms are not clear. Given their importance in cellular metabolism and regulatory processes, these therapeutic agents are expected to target distinct pathways either directly or indirectly. Therefore, identification of the targets of these drugs might assist in characterizing their molecular side effects.

Molecular changes accompanying the progression of disease can now be determined by several approaches. Gene expression microarray analysis has been widely used during the past decade for characterizing complete transcriptomes [7–9] and it has yielded global profiles of whole retina or retinal cell types in both wild type and disease models [10–18]. A comparison of the expressed complement of the genome between normal and diabetic retinas has indicated altered abundance of transcripts involved in several key pathways [19,20]. Although microarray strategies have been successful in describing disease- or phenotype-associated expression changes, hybridization-based profiling approaches suffer from technical variations that are difficult to control. For this reason, many expression changes cannot be validated by quantitative reverse transcription polymerase chain reaction (qRT–PCR) [21]. In addition, relevant causative expression changes, such as alternatively spliced variants of transcripts and the expression of novel transcripts in disease samples, may not be comprehensively captured because specific probe sets may not be included on the particular microarrays being used.

Next generation sequencing based on the RNA sequencing (RNA-seq) approach is now gaining prominence as a means of accurate qualitative and quantitative characterization of the expressed complement of a genome [22,23]. This method provides millions of sequences from expressed RNA molecules and can provide relatively unambiguous definition and abundance of transcripts in a given specimen. RNA-seq is therefore expected to reveal a better representation of the transcriptome, and this strategy is also more amenable for the analysis of alternatively spliced transcripts.

We have recently demonstrated the high accuracy and sensitivity of RNA-seq technology with microarray and qRT–PCR methods by profiling the neural retina specific leucine zipper deficient (*Nrl^−/−^*) retina [24]. In this paper, we define the changes occurring in the entire retinal transcriptome of streptozotocin (STZ)-induced diabetic mice compared to wild type mice. We also attempt to identify relevant molecular signatures and cellular pathways, with the goal of examining the impact of inhibitors of RAGE and of p38 MAP kinase, two candidate drugs that inhibit retinopathy in experimental models. Our results indicate that these inhibitors induce common, as well as unique, molecular changes that may assist in determining the efficacy and/or side effects of candidate drugs.

## Methods

### Streptozotocin-induced diabetes in mice

All animal experiments conformed to the ARVO Resolution on the Use of Animals in Research and were approved by the Animal Care and Use Committee of Case Western University. Diabetes was induced in C57BL/6 mice (Jackson Laboratories, Bar Harbor, ME) with streptozotocin and a corresponding number of weight and age-matched animals were maintained as normal controls. Mice received five sequential daily intraperitoneal injections of a freshly prepared solution of streptozotocin (Sigma-Aldrich, St Louis, MO) in citrate buffer (pH 4.5) at 60 mg/kg of bodyweight. After hyperglycemia was verified at least three times during the second week after streptozotocin administration, diabetic mice were randomly assigned to remain as untreated diabetic controls or were administered RAGE-Ig fusion protein intraperitoneally at three different concentrations (10, 100, and 300 µg per mouse) three times per week. Insulin was given to diabetics as needed (0–0.3 units of neutral protamine Hagedorn (NPH) insulin subcutaneously, 0–3 times per week) to prevent weight loss without preventing hyperglycemia.

The RAGE fusion protein (provided by L. Brown, Galactica Pharmaceuticals, Inc., Villanova, PA) consists of a RAGE ligand binding element, a heavy chain immunoglobulin of G4 isotype (IgG4) constant domain with a linker connecting the ligand binding element with the constant domain. The RAGE-Fc binds to all of the known ligands of RAGE and acts as a competitive, negative regulator of RAGE signaling by competing with the membrane-bound receptor for binding of ligands. For the present studies, a murine version of hRAGE was employed in which a G2a isotype (IgG2a) constant domain (biologically equivalent to IgG4 in hRAGE) was used. The inhibitor of p38 MAPK (PHA666859) was provided by Pfizer Research Laboratories (Groton, CT). PHA666859 was mixed into powdered diet and replaced weekly, and food consumption was measured to calculate the amount of drug consumed.

We have previously shown that both of these therapies inhibit the development of early stages of diabetic retinopathy, as determined by the abundance of acellular capillaries and pericyte ghosts [3,25]. Glycated hemoglobin (GHb; an estimate of the average level of hyperglycemia over the previous 2–3 months) was measured by affinity chromatography (Glyc-Affin; Pierce, Rockford, IL) every 3 months in each animal, after an overnight fast . Bodyweight and average daily food consumption were measured weekly. Experimental variations were reduced by CO_2_ asphyxiation of all animals at 8 months of diabetes between 2 – 4 PM

### Isolation of RNA

Retinal RNA was extracted with Trizol reagent [26,27]. Three independent retina samples were obtained from groups of animals that were normal, diabetic, treated with RAGE inhibitor, and treated with p38 MAPK inhibitor. The RNA was quantified by absorbance at 260 nm in a spectrophotometer and its integrity was determined using an Agilent Bioanalyzer (Santa Clara, CA) [28].

### Illumina RNA-seq

Independent RNA samples were used for the preparation of cDNA libraries using an mRNA-seq Sample Preparation Kit (Illumina, San Diego, CA). Briefly, double stranded cDNA was prepared by random hexamer priming and reverse transcription of chemically sheared total RNA (2 µg). The double stranded cDNA was ligated with adaptor sequences required for bridge amplification using the Illumina flow-cell, and the ligated DNA was amplified before library assessment and quantitation using the BioAnalyzer DNA-1000 chip (Agilent). Cluster generation of 10 pM loads of a single mRNA-seq library per lane was performed on the Cluster Station using an Illumina Single Read Cluster Generation Kit v4 (Illumina). Sequence-by-synthesis single reads of 54 base length using the SBS Sequencing Kit v4 (Illumina) were generated on the Genome Analyzer IIx. This instrument runs real-time analysis SCS2.4 software (Illumina) for base calling.

### Real-time qPCR

Quantitative RT–PCR was performed using a Genetic Analyzer 7900HT (Life Technologies, Carlsbad, CA) with Taqman or SYBR green assays, as previously described [24]. Briefly, total RNA (1 µg) was reverse transcribed by Superscript II reagents (Life Technologies, Carlsbad, CA) primed with oligo(dT)_20_. The cDNA was diluted fivefold and one microliter was used as template in subsequent qRT–PCR reactions. The integrity of the PCR reaction was verified by melt curve analysis. The results of amplifications of three biologic replicates, performed in triplicate, were averaged to produce the qRT–PCR data. Changes in gene expression were determined by the ddCT method using the levels of *HPRT* (hypoxanthine guanine phosphoribosyl transferase) transcript for normalization.

### Bioinformatics analysis of RNA-seq data

The cDNA sequences captured on the Illumina platform were analyzed using the following workflows.

(1) Transcript isoform level analysis was performed by aligning the 54 base cDNA reads against the reference genome mm9 build using a Burrows-Wheeler transform based short read aligner (BWA) [29], and the aligned reads were visualized using Integrated Genome Viewer [30]. The read alignment files were imported into Partek Genomics Suite (Partek® Genomics SuiteTM. 6.3 ed. St. Louis, MO: Partek Inc.; 2008) and RPKM (reads per kilobase of exon model per million mapped reads) counts for each of the 28,157 transcripts defined in the UCSC refflat annotation file were calculated. A stringent filtering criterion with RPKM value of 1.0 [31] was used to obtain 16,577 expressed transcripts. The RPKM values of filtered transcripts were log-transformed using log_2_ (RPKM + offset) with an offset value of 1.0. This filtering protocol reduced the number of transcripts from 28,157 to 16,577 for further analyses. Fold changes in transcript expression and *p*-values were computed using ANOVA, and significantly altered transcripts were selected by applying fold change-cutoff of 1.5 and/or p-value cutoff of 0.05. Gene Ontology (GO) pathways that were significantly enriched between each of the sample pairs were identified using ExPlain tool from Biobase International, Beverly, MA.

(2) Alternatively, gene level analysis was performed by aligning the filtered reads to the mouse reference genome build mm9 using the ELAND (Efficient Local Alignment of Nucleotide Data) algorithm (Anthony J Cox, Solexa Ltd Saffron, Walden, UK). First, raw and RPKM normalized counts were calculated for gene models (as defined in UCSC RefGene). Subsequently, we applied SAM (significance analysis of microarrays) to gene level RPKM to identify RNAs with a fold change greater than 1.5 and false discovery rate (FDR) of less than 25%. Briefly, the boundaries of exons were obtained from the RefGene database (Genome), and the numbers of mapped reads on each exon were calculated. The mapped reads as RPKM indicated the expression level of exons, and this value was used to calculate the Log base 2 of the average (or maximum) expression level of exons for each gene. For this alternative analysis, a less stringent cutoff of 0.5 RPKM was used to select expressed genes for further analysis.

### Alternatively spliced exons

Alternatively spliced transcripts were detected using the AltAnalyze tool [32], which employs de novo splice junctions to predict alternative splicing events based on the reads spanning the splice junctions. First, TopHat [33] was run independently on sequence reads obtained for each of the 12 RNA samples to identify all novel splice junctions. Second, we combined all predicted splice junctions into a master file and used this as an input option, together with the Ensembl mouse gene transfer format (GTF) annotation file, for a second iteration of TopHat runs on each of the 12 samples. Finally, these 12 junction files were used as input in AltAnalyze to detect alternative splicing using the analysis of splicing by isoform reciprocity (ASPIRE) algorithm.

Briefly, we calculated the number of sequences mapping to a specific exon junction and the numbers of sequences mapping to junctions as well as exons of that specific gene. These two numbers were then used to calculate the fraction of the junction containing sequences relative to the mean of abundance of sequences of all junctions and exons in the gene. The procedure was applied to each reciprocal isoform pair (i.e., an isoform that includes a specific exon and an isoform in which that exon is excluded). The inclusion and exclusion ratios were then used to compute the value for ΔI. The details of these protocols have been described in AltAnalyze workflow [32], as adapted from ASPIRE algorithm [34]. The ratios described above were used to calculate p values for the sample ASPIRE scores relative to controls, and to determine false-discovery rate p values [35].

## Results

### Glycemia

Diabetic mice from all experimental groups had levels of GHb and blood glucose that were significantly greater (p<0.05) than levels found in appropriately age-matched nondiabetic controls. Average GHb for the nondiabetic control (N), diabetic control (D), diabetic plus PHA666859 (D + PHA666859), and diabetic plus murine RAGE-Fc fusion protein (D + mRAGE-Fc) groups over the entire duration of study are listed in Table 1. Although diabetic mice did not gain weight at the normal rate, none of the animals lost bodyweight, and all of the animals appeared clinically healthy. These results indicate that the therapies involving inhibitors of RAGE and p38 MAP kinase do not alter the diabetic status of the animals.

**Table 1:** Glycemic status of control and drug-treated animals over the 8 months of the study.

Control/Treatments*	**Glucose**	**Glycated hemoglobin (GHb)**
Nondiabetic Controls (N)	130+8	3.0+0.1
Diabetic Controls (D)	400+21	10.4+0.9
Diabetic plus p38 MAPK inhibitor (D+PHA666859)	411+32	10.3+0.6
Diabetic plus murine RAGE-Fc Fusion Protein (D + mRAGE-Fc)	393+31	10.6+0.7

*The values are reported as mean ± standard deviation, and were calculated for 10, 7, 7, and 8 animals in the four groups, respectively.

### Raw read counts and transcripts in sequenced samples

Total raw reads for various samples were in the range of 27.8–35.5 million, which represented ~1.6 gigabases of sequences for each sample. Efficient Local Alignment of Nucleotide Data (ELAND) and Burrows-Wheeler Alignment (BWA) tools allowed the alignment of 13.8- 20.9×10^6^ reads from various samples to mouse genome sequence database (build mm9). These aligned reads were subsequently analyzed by significance analysis of microarrays (SAM) or ANOVA. The sequence alignment files were imported into PARTEK, and the normalized values for numbers of RPKM for 28,157 transcripts defined in the UCSC refflat file were obtained. Among these sequences obtained from nondiabetic and diabetic animals, 16,231 transcripts had RPKM values of greater than one, and included rhodopsin (*Rho*), guanine nucleotide binding protein (*Gnat1*), retinol binding protein (*Rbp1*), phosphodiesterase 6A *(Pde6a*), and guanine nucleotide binding protein (*Gngt1*) as the most abundant sequences specific to photoreceptor cells. Similarly, the list also contained other less abundant transcripts such as paired-like homeodomain 3 (*Pitx3*), PDZ and LIM domain 1(*Pdlim1*), and meteorin (*Metrnl*). The presence of these highly abundant as well as rare transcripts in the data set demonstrates that the library preparation and data analysis methods did not introduce any undesirable bias.

### Comparison of transcript differences revealed by two analyses

**Figure 1:**
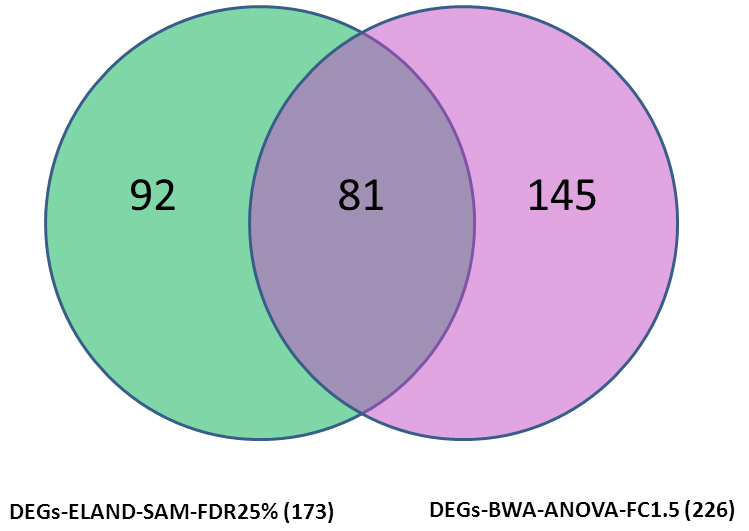
Differentially expressed gene sets were obtained by employing either significance of microarray analysis algorithm or ANOVA method. The gene sets were applied as described in Methods section and the genes unique and common in the two sets are indicated.

We analyzed the sequence data using ELAND-SAM and BWA-ANOVA methods, and compared the results to confirm method-specific changes in transcript abundance in the various groups of animals. Differential expression in diabetic and normal animals revealed the top 100 transcripts shortlisted by the two methods, as shown in Table 2 (Appendix 1). Of these 100 transcripts, 81 are present in both data sets (Figure 1) including a variety of crystallin genes. However, UDP-glucuronosyl transferase transcripts were detected only by the ELAND-SAM method. A significant difference was also noted in the fold-changes indicated by the two methods. These differences can be attributed to differences in the alignment algorithms, filtering criteria, and calculation of differential gene expression between the two methods, and demonstrate that altered abundance of transcripts can be detected by either one of these two methods.

**Table 2:** Comparison of transcript abundance changes obtained by two methods*

**Gene**	Fold-method I*	**Gene**	Fold-method I*	**Gene**	Fold-method II*	**Gene**	Fold-method II*
Crybb2	19.8	Tmem40	2.4	Rnu73a	93.3	Pdlim1	3.7
Cryba1	19.4	Crygc	2.4	Lim2	75.7	Trim16	3.7
Cryba4	18.3	Acta2	2.3	Wnt7a	47.9	Lenep	3.6
Crygs	18.2	Igfbp7	2.3	Pitx3	40.1	S100a6	3.6
Cryaa	17.9	Lyz2	2.3	Tmem40	38	Cd44	3.6
Lim2	17.6	Vit	2.3	Mip	36	Gpd1	3.5
Mip	16.7	Tmem27	2.2	Wnt7b	26.1	Cbr2	3.4
Cryba2	16.4	Gsn	2.2	Gja3	23.8	Nanos2	3.4
Crybb1	15.1	C1qtnf5	2.2	Grifin	23.7	Gpnmb	3.4
Bfsp2	10.7	Mfrp	2.2	Cryba4	23.1	Gsta3	3.4
Lgsn	10.1	Igfbp7	2.1	Bfsp2	21	Ugt1a5	3.3
Grifin	9.8	Ucp2	2.1	Crybb2	20.3	Ugt1a10	3.3
Crybb3	9.2	Rspo1	2.1	Crybb1	20.2	Ugt1a1	3.3
Crybb3	8.9	Crygc	2.1	Crygs	20	Ugt1a9	3.3
Dct	7.2	Wnt7a	2.1	Cryba1	19.8	Ugt1a2	3.3
Cd24a	6.7	Trpm1	2.1	Cryaa	18.9	Bmper	3.3
Bfsp1	6.6	Npff	2.1	Cryba2	18.5	Ugt1a7c	3.3
Lctl	6.6	Krt15	2.1	Tyr	18.5	Ugt1a6a	3.2
Cryab	6.6	Selenbp1	2.1	Crybb3	16.3	1700093K21Ri	3.2
S100a4	4.9	Tspan10	2	Lctl	14.5	1110017D15Ri	3.1
Ttr	4.8	Mgst1	2	Cd24a	10.7	Ugt1a6b	3.1
Mlana	4.8	Tmem176a	2	Mlana	10.7	Cox8b	3.1
Gja3	4.6	Papss2	2	Dct	9.8	Acta2	3.1
Crygb	4.5	Mlph	2	Krt5	9.2	Pla2g2f	3
Rgr	4.2	Gsta3	2	Sfrp1	7.8	Ahnak	3
Crygd	3.9	Gsto1	2	Lrat	6.8	Lix1	2.9
Pmel	3.9	Perp	2	S100a4	6.4	Adamts4	2.8
Sfrp1	3.9	Srd5a2	2	Ttr	6.4	Ela1	2.7
Egr1	3.6	Psca	2	Mlph	6.3	Angptl4	2.7
Tyrp1	3.3	C1qtnf5	2	Rgr	5.7	Galr2	2.6
Aldh3a1	3.3	Mfrp	2	Tgm1	5.6	Dock5	2.6
Fabp5	3.2	Lgals1	1.9	Rpe65	5.5	Scel	2.6
Lenep	3.2	Islr	1.9	Emp1	5.3	Ppfibp2	2.6
Gpnmb	3.2	Penk	1.9	Srd5a2	5.2	Pld5	2.6
Gja8	3.2	Oca2	1.9	Slc6a20a	5.2	Thbs1	2.5
Serpina3n	3.1	Aldh3a1	1.9	Fam46c	5	Selenbp1	2.5
Wnt7b	2.9	Ak1	1.9	D21Rik	4.8	Ucp2	2.5

*The work-flow, Method I (BWA-ANOVA) and Method II (ELAND-SAM) are described in the Methods’ section. The numbers indicate fold changes between diabetic and nondiabetic controls as determined by two different algorithms.

### Validation of significantly altered transcripts

**Figure 2:**
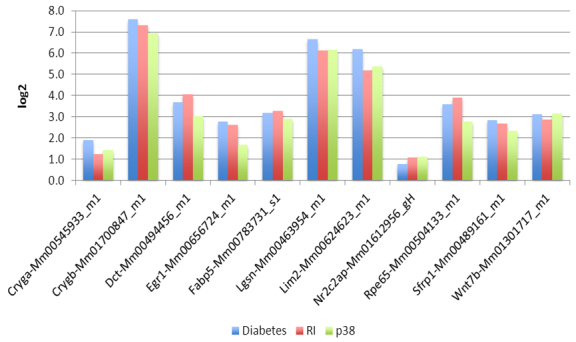
TaqMan real-time quantitative polymerase chain reaction (qPCR) validation was performed for selected transcripts. The transcripts were amplified as described in Methods section. Three technical replicates were performed for three biologic replicate samples of RNA isolated from nondiabetic animals, diabetic animals and diabetic animals treated with either the inhibitor of receptor for advanced glycation endproducts (RAGE) indicated as RI, or the inhibitor of p38 mitogen activated protein kinase (MAPK) designated as p38. The bars (left to right) represent samples corresponding to diabetic animals, diabetic animals treated with RAGE inhibitor, and diabetic animals treated with p38 MAPK inhibitor, respectively. Fold increase was calculated with respect to nondiabetic controls.

Some representative transcripts were chosen for validation based on the magnitude of the changes in their abundance and their involvement in various pathways. We performed Taqman real-time PCR to validate 11 transcripts, including *Cryga*-crystallin gamma A, *Crygb*-crystallin gamma B, *Dct*-dopachrome tautomerase, *Egr*-early growth response, *FABP*-fatty acid binding protein, *Lgsn*-lengsin, *Lim2*-lens intrinsic membrane protein, *Nr2c2ap*-nuclear receptor 2C2-associated protein, retinal pigment epithelium 65 K_d_ protein (*Rpe65*), *Sfrp1*-secreted frizzled-related protein, and wingless type MMTV integration site, member 7b (*Wnt7b*). The level of each of these transcripts corresponded to the pattern revealed by RNA-seq, and indicated significant changes in the levels of these transcripts between diabetic and normal mice (Figure 2). However, the magnitude of change observed by RT–PCR was not identical to the results of RNA-seq. The non-correspondence of magnitude reflects the differences in sensitivities of the RNA-seq and Taqman assays, which are two inherently different strategies.

**Figure 3:**
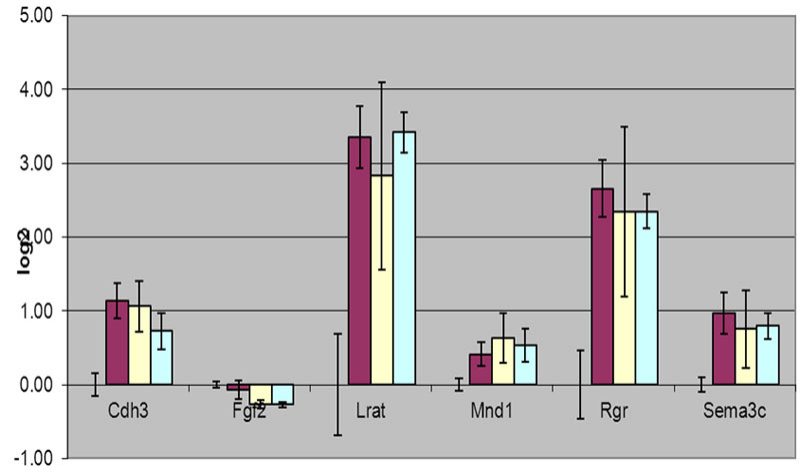
Cyanine (SYBR) green real-time quantitative polymerase chain reaction (qPCR) validation was performed for selected transcripts. The transcripts were amplified as described in Methods section. Three technical replicates were performed for three biologic replicate samples of RNA isolated from nondiabetic animals, diabetic animals and diabetic animals treated with either inhibitor of receptor for advanced glycation endproducts (RAGE) indicated as RI, or an inhibitor of p38 mitogen activated protein kinase (MAPK) designated as p38. The bars (left to right) represent samples corresponding to diabetic animals, diabetic animals treated with RAGE inhibitor, and diabetic animals treated with p38 MAPK inhibitor, respectively. The error bars represent standard deviation from the mean value (n=9). The levels of transcript in diabetic and treated animals were determined relative to the levels present in nondiabetic controls.

A few additional transcripts (*Cdh3*-cadherin, *Fgf2*-fibroblast growth factor, *Lrat*-lecithin retinol acyltransferase, *Mnd1*-meiotic nuclear divisions 1 homolog, *Rgr*-retinal G-protein coupled receptor, and *Sema3c*-semaphorin) were chosen for validation with SYBR green based real-time qPCR. All of these transcripts were significantly altered in diabetic mice (Figure 3). As stated for Taqman qPCR validation, the correspondence between RNA-seq and SYBR green qPCR was comparable but not identical.

### Transcripts, pathways and gene ontology (GO) categories are altered in retinas isolated from diabetic animals

The application of SAM and a false discovery rate of 25% for the data set revealed greater than 1.5 fold alterations in the levels of 173 transcripts. Alternative analysis with BWA-ANOVA, however, indicated alterations of greater than 1.5 fold in 298 transcripts. Among several GO categories, significantly altered transcripts revealed by either of the two methods were grouped together as structural constituents of eye lens, visual perception, sensory perception of light stimulus, eye development, structural molecule activity, microsomes, the vesicular fraction, and glucuronosyl transferase activity. Changes were also observed in transcripts involved in inflammatory pathway, apoptosis, microvasculature, Wnt signaling, photoreceptors, and neural cell function. The alterations in the levels of these transcripts are described below.

### Crystallins, a major class of retinal genes, are altered in diabetic retinopathy

More than 20 variants of crystallins have been detected in the retina. Of these, 13 transcripts were increased by greater than 1.6 fold (Table 3). The levels of *Cryaa*, *Cryba1*, *Cryba2*, *Cryba4*, *Crybb1*, *Crybb2*, and *Crygs* in diabetic animals were elevated by >9.0 fold, whereas the abundance of *Cryab*, *Crygb*, *Crygc*, *Crygd*, and *Crygn* in diabetic animals varied between 1.6 and 6.6 fold compared to non-diabetic controls. These transcript changes were detected by both analysis methods.

**Table 3:** Alterations in the levels of crystallin transcripts in diabetic retinopathy and the reversal of their levels by RAGE inhibitor and p38 MAPK inhibitor.

		Fold change*	Fold change*	Fold change*
**Gene symbol**	**Gene name**	**D vs N**	**D + mRAGE-Fc vs N**	**D + PHA666859 vs N**
Crybb2	Crystallin, Beta B2	19.8	3.6	2.4
Cryba1	Crystallin, Beta A1	19.3	3.7	1.9
Cryba4	Crystallin, Beta A4	18.3	3.8	2.4
Crygs	Crystallin, Gamma S	18.2	3.9	2.5
Cryaa	Crystallin, Alpha A	17.9	3.5	2.4
Cryba2	Crystallin, Beta A2	16.4	3.4	2.1
Crybb1	Crystallin, Beta B1	15.1	3.9	2.8
Crybb3	Crystallin, Beta B3	9.2	3.2	2.5
Cryab	Crystallin, Alpha B	6.6	3.6	1.9
Crygb	Crystallin, Gamma B	4.5	3.2	2.2
Crygd	Crystallin, Gamma D	3.9	2.6	2.3
Crygc	Crystallin, Gamma C	2.4	1.7	1.6
Crygn	Crystallin, Gamma N	1.6	1.5	1.2

*D=diabetic control, n=nondiabetic control, D+mRAGE-Fc=diabetic plus murine RAGE-Fc fusion protein, D+PHA666859=diabetic plus p38 MAPK inhibitor PHA666859.

### Alterations in Wnt signaling transcripts

Altered levels were observed for 14 transcripts that are either directly involved in Wnt signaling pathway or are targets of Wnt signaling (Table 4). While *Sfrp1* and *Wnt7b* levels in diabetic animals increased by 3.9 and 2.9 fold, *Wnt7a* and *Mfrp* were upregulated by more than 2.0 fold. The increase in the levels of *Wnt5b* and *axin*, and decrease in *Wisp 1* and *Wnt2b* transcripts were marginal. The levels of a few target genes of Wnt signaling such as *FRAT2*, *BMP4*, *Cldn1*, and *Cldn2* were also altered.

**Table 4:** Alterations in the levels of transcripts involved in Wnt signaling pathway.

		Fold change*	Fold change*	Fold change*
**Gene symbol**	**Gene name**	**D vs N**	**D + mRAGE-Fc vs N**	**D + PHA666859 vs N**
Sfrp1	Secreted frizzled-related protein 1	3.9	2.9	1.9
Wnt7b	Wingless-related MMTV integration site 7B	2.9	1.9	1.5
Wnt7a	Wingless-related MMTV integration site 7A	2.1	1.5	1.5
Mfrp	Membrane-type frizzled-related protein	2.1	1.9	1.5
Cldn1	Claudin 1	1.6	1.4	1.4
Cldn2	Claudin 2	1.6	1.4	1.6
BMP4	Bone morphogenetic protein 4	1.5	1.6	1.5
Id2	Inhibitor of DNA binding 2	1.4	1.6	1.4
Axin1	Axin 1; axis inhibition protein 1	1.3	1.2	1.6
FRAT2	Frequently rearranged in advanced T-cell lymphomas 2	1.2	1.4	1.8
Wnt5b	Wingless-related MMTV integration site 5B	1.2	1.3	1.4
Wisp1	WNT1 inducible signaling pathway protein 1	1.5 (down)	1.6 (down)	2.4 (down)
Wnt2b	Wingless related MMTV integration site 2b	1.1 (down)	1.1 (down)	1.05 (down)

*D=diabetic control, n=nondiabetic control, D+mRAGE-Fc=diabetic plus murine RAGE-Fc fusion protein, D+PHA666859=diabetic plus p38 MAPK inhibitor PHA666859.

### Transcripts involved in microvasculature and the inflammatory pathway

Endothelin and *VEGF* were among the vascular transcripts detected in the retina. The levels of *Edn2* decreased by 1.4 fold, whereas an increase of 1.4 fold was observed in the levels of *Edn3* (Table 5). The levels of other vasculature related genes were marginally altered in diabetic animals. Alterations were also observed in the abundance of inflammation associated transcripts, namely *Ltbp1*, *Bmp4*, *Hspb1*, *CD44*, *C1qtnf5*, *Ifitm1*, and *Islr*, which varied between 1.5 and 2.2 fold in diabetic animals (Table 6).

**Table 5:** Altered levels of microvasculature transcripts associated with diabetic retinopathy.

		Fold change*	Fold change*	Fold change*
**Gene symbol**	**Gene name**	**D vs N**	**D + mRAGE-Fc vs N**	**D + PHA666859 vs N**
Edn3	Endothelin 3	1.4	1.5	1.4
VEGFB	Vascular endothelial growth factor B	1.1	1.3 (down)	1.1 (down)
VEGFA	Vascular endothelial growth factor A	1.02	1.4	1.3
Edn2	Endothelin 2	1.4 (down)	1.6 (down)	2.0 (down)

*D=diabetic control, n=nondiabetic control, D + mRAGE-Fc=diabetic plus murine RAGE-Fc fusion protein, D + PHA666859=diabetic plus p38 MAPK inhibitor PHA666859.

**Table 6:** Relative levels of transcripts associated with inflammatory pathway in diabetic mice.

		Fold change*	Fold change*	Fold change*
**Gene symbol**	**Gene name**	**D vs N**	**D + mRAGE-Fc vs N**	**D + PHA666859 vs N**
C1qtnf5	C1q and tumor necrosis factor related protein 5	2.2	1.9	1.5
CD44	CD44 antigen	1.7	1.3	1.2
Ltbp1	Latent transforming growth factor beta binding protein 1	1.6	1.4	1.4
Islr	Immunoglobulin superfamily containing leucine-rich repeat	1.6	1.5	1.6
Hspb1	Heat shock protein 1	1.6	1.6	1.2
Ifitm1	Interferon induced transmembrane protein 1	1.5	1.4	1.3
Bmp4	Bone morphogenetic protein 4	1.5	1.6	1.5

*D=diabetic control, n=nondiabetic control, D+mRAGE-Fc=diabetic plus murine RAGE-Fc fusion protein, D+PHA666859=diabetic plus p38 MAPK inhibitor PHA666859.

### Transcripts involved in apoptosis

We analyzed a variety of pro-apoptotic and anti-apoptotic transcripts as well as other transcripts that influence the apoptosis pathways. A significant increase was observed in the levels of *Dapl1* and *Bid1*, while the levels of *Fads3*, *Fas*, and *Bag5* showed marginal alterations. An appreciable decrease was also noted in the levels of *Trafip3*, *Tnfsf13*, *Tnfrsf18*, and *Casp7* (Table 7). It warrants mention that these transcripts were not shortlisted when the FDR and *p*-value cut-off were applied to the data analysis. Given the importance of apoptosis in diabetic retinopathy [36,37], these genes were specifically analyzed from the master list of sequenced tags and were found to have altered levels in the diabetic animals.

**Table 7:** Changes in the levels of transcripts involved in apoptosis pathway.

		Fold change*	Fold change*	Fold change*
**Gene symbol**	**Gene name**	**D vs N**	**D + mRAGE-Fc vs N**	**D + PHA666859 vs N**
Dapl1	Death associated protein-like 1	2.6	1.2	1.2
Bid	BH3 interacting domain death agonist	1.6	1.5	1.3
Fads3	Fatty acid desaturase 3	1.3	1.2	1.2
Fas	Fas (TNF receptor superfamily member 6)	1.3	1.1	1.2
Bag5	BCL2-associated athanogene 5	1.2	1.2	1.4
Traf3ip3	TRAF3 interacting protein 3	1.5 (down)	1.5 (down)	2.5 (down)
Tnfsf13	Tumor Necrosis Factor superfamily member 13	1.4 (down)	1.5 (down)	1.6 (down)
Tnfrsf18	Tumor necrosis factor receptor superfamily, member 18	1.4 (down)	1.7 (down)	2.2 (down)
Casp7	Caspase 7	1.4 (down)	1.5 (down)	2.0 (down)
Bcl7c	B-cell CLL/lymphoma 7C	1.1 (down)	1.6 (down)	1.3 (down)

*D=diabetic control, n=nondiabetic control, D+mRAGE-Fc=diabetic plus murine RAGE-Fc fusion protein, D+PHA666859=diabetic plus p38 MAPK inhibitor PHA666859.

### Transcripts involved in neuronal functions and photoreceptors

The list of differentially expressed genes obtained by applying stringent threshold parameters did not contain any transcripts involved in neuronal development or signal transduction. However, a review of the transcript master list, without applying rigorous statistical significance, revealed changes in transcripts encoded by polycomb genes (*Pcgf*), acetyl choline receptor genes (*Chrn*), muscarinic cholinergic receptor genes (*Chrm*), potassium channels (*Kcnq*), glutamate receptor genes (*Grin*), solute carrier/transporter genes (*Slc*), and the fasciculation and elongation gene (*Fez*) (Table 8). Similar review revealed decreased levels of transcripts involved in the development and function of photoreceptors. These transcripts included cyclic nucleotide gated channel (*Cngb3*), arrestin (*Arr*), guanine nucleotide binding protein (*Gnb3*), and phosphodiesterase (*Pde6h*). Marginal decreases were also noticed in photoreceptor-specific transducin (*Gnat2*) and *Crxos1*. However, the levels of *Nrl*, *Crx*, and *Nr2e3* remained relatively unchanged (Table 9).

**Table 8:** Altered levels of transcripts involved in neuronal functions

		Fold change*	Fold change*	Fold change*
**Gene symbol**	**Gene name**	**D vs N**	**D + mRAGE-Fc vs N**	**D + PHA666859 vs N**
Pcgf2	Polycomb group ring finger 2	1.5	1.3	1.2
Slc15a2	Solute carrier family 15 (H^+^/peptide transporter), member 2	1.3	1.1	1.1
Kcnq4	Potassium voltage-gated channel, subfamily Q, member 4	1.2	1.1	1.1 (down)
Chrna2	Cholinergic receptor, nicotinic, alpha polypeptide 2 (neuronal)	1.2	1.2	1.2
Grinl1a	Glutamate receptor, ionotropic, NMDA like 1A	1.2	2	2
Chrm4	Cholinergic receptor, muscarinic 4	1.04	1.2	1.4
Slc6a5	Solute carrier family 6 (neurotransmitter transporter, glycine), member 5	1	1.39 (down)	1.32 (down)
Kcnip4	Kv channel interacting protein 4	1.25 (down)	1.13 (down)	1.62 (down)
Kcnma1	Potassium large conductance calcium-activated channel, subfamily M, alpha member 1	1.24 (down)	1.62 (down)	1.57 (down)
Grin1	Glutamate receptor, ionotropic, NMDA1 (zeta 1)	1.21 (down)	1.79 (down)	1.47 (down)
Chrnb3	Cholinergic receptor, nicotinic, beta polypeptide 3	1.08 (down)	1.44 (down)	1.35 (down)
Fez1	Fasciculation and elongation protein zeta 1 (zygin I)	1.03 (down)	1.35 (down)	1.29 (down)

*D=diabetic control, n=nondiabetic control, D+mRAGE-Fc=diabetic plus murine RAGE-Fc fusion protein, D+PHA666859=diabetic plus p38 MAPK inhibitor PHA666859.

**Table 9:** Altered levels of selected photoreceptor-specific transcripts

		Fold change*	Fold change*	Fold change*
**Gene symbol**	**Gene name**	**D vs N**	**D + mRAGE-Fc vs N**	**D + PHA666859 vs N**
Cngb3	Cyclic nucleotide gated channel beta 3	1.3 (down)	1.4 (down)	1.4 (down)
Arr3	Arrestin 3, retinal	1.3 (down)	1.6 (down)	1.8 (down)
Gnb3	Guanine nucleotide binding protein (G protein), beta 3	1.3 (down)	1.7 (down)	1.8 (down)
Pde6h	Phosphodiesterase 6H, cGMP-specific, cone, gamma	1.2 (down)	1.8 (down)	2.2 (down)
Pde6a	Phosphodiesterase 6A, cGMP-specific, rod, alpha	1.2 (down)	1.4 (down)	1.5 (down)
Gnat2	Guanine nucleotide binding protein, alpha transducing 2	1.1 (down)	1.3 (down)	1.2 (down)
Crxos1	Crx opposite strand transcript 1	1.1 (down)	1.4 (down)	1.7 (down)
Pde6c	Phosphodiesterase 6C, cGMP specific, cone, alpha prime	1.1 (down)	1.7 (down)	1.8 (down)
Nrl	Neural retina leucine zipper gene	1.02 (down)	1.1	1.1
Crx	Cone-rod homeobox containing gene	1.03	1.3	1.2
Nr2e3	Nuclear receptor subfamily 2, group E, member 3	1.01	1.11 (down)	1.22 (down)

*D=diabetic control, n=nondiabetic control, D+mRAGE-Fc=diabetic plus murine RAGE-Fc fusion protein, D+PHA666859=diabetic plus p38 MAPK inhibitor PHA666859.

### Altered expression of genes encoding glucuronosyl transferases in diabetic retinopathy

A significant increase was observed in transcripts for members of the UDP-glucuronosyl transferase gene family during diabetic retinopathy. The protein products of these genes are primarily responsible for the metabolism of xenobiotics. In particular, *UGT1A5*, *UGT1A10*, *UGT1A1*, *UGT1A9*, *UGT1A2*, *UGT1A7C*, *UGT1A6A*, and *UGT1A6B* transcripts were elevated by greater than 3.0 fold in diabetic animals (Table 10). These transcripts were detected in analyses performed by the ELAND-SAM method at the gene level with a FDR cutoff of 25%, but were absent in the list of transcripts generated by transcript isoform level BWA-ANOVA analysis at a p value cutoff of less than 0.05.

**Table 10:** Changes in the abundance of UDP glucuronosyl transferase transcripts in diabetic animals

		Fold change*	Fold change*	Fold change*
**Gene symbol**	**Gene name**	**D vs N**	**D + mRAGE-Fc vs N**	**D + PHA666859 vs N**
Ugt1a5	UDP glucuronosyltransferase 1 family, polypeptide A5	3.3	2	1.5
Ugt1a10	UDP glucuronosyltransferase 1 family, polypeptide A10	3.3	2	1.5
Ugt1a1	UDP glucuronosyltransferase 1 family, polypeptide A1	3.3	2	1.5
Ugt1a9	UDP glucuronosyltransferase 1 family, polypeptide A9	3.3	2	1.5
Ugt1a2	UDP glucuronosyltransferase 1 family, polypeptide A2	3.3	2	1.5
Ugt1a7c	UDP glucuronosyltransferase 1 family, polypeptide A7C	3.3	1.9	1.5
Ugt1a6a	UDP glucuronosyltransferase 1 family, polypeptide A6A	3.3	2	1.5
Ugt1a6b	UDP glucuronosyltransferase 1 family, polypeptide A6B	3.1	2.1	1.5

*D=diabetic control, n=nondiabetic control, D+mRAGE-Fc=diabetic plus murine RAGE-Fc fusion protein, D+PHA666859=diabetic plus p38 MAPK inhibitor PHA666859.

### Rhodopsin-retinol pathway altered in diabetic mice

Rhodopsin-retinol pathway transcripts showed increased levels for lecithin retinol acetyl transferasease, transthyretin, retinol binding protein, and *Stra6* (stimulated by retinoic acid). However, decreases occurred in the levels of transcripts corresponding to retinoic acid metabolism enzyme *Cyp26a1*, retinol dehydrogenase, and rhodopsin phosphorylating GPCR kinase (*Grk4*).

### Pathways related to altered metabolism in diabetic mice

Transcripts directly/indirectly related to glycemic pathway showed marginal increases in the case of glycerol-3-phosphate dehydrogenase and insulin secretion modulating *Trpm4* protein, whereas transcripts levels decreased slightly for a glycogen interacting protein *Trim7* and for insulin-like growth factor binding proteins *Igfbp3* and *Igfbp5*.

### Alternative splicing of genes in diabetic retinopathy

The RNA-seq method provides the identity of transcripts that have yielded an unambiguous sequence. Some of the sequence reads, which span exon boundaries, can be analyzed to predict alternatively spliced transcripts. We identified 55 genes that were alternatively spliced in the retina of diabetic animals (Table 11). The alternative splicing events in these genes were detected by selecting transcripts with a RPKM cutoff of 3.0 and using ASPIRE’s ΔI cutoff of 0.2, which indicates a 20% change in exon inclusion. This threshold of ΔI has been shown to yield accurate representations of alternatively spliced versions of transcripts [34].

**Table 11:** Alternatively spliced transcripts in diabetic animals

**Gene symbol**	**Gene name**	**Gene symbol**	**Gene name**
Abca8a	ATP-binding cassette	Agtpbp1	ATP/GTP binding protein
Ankrd39	ankyrin repeat domain	Ank2	ankyrin 2, brain
Apip	APAF1 interacting	Dus1l	dihydrouridine synthase
Bdh1	3-hydroxybutyrate dehydrogenase	Eif4g3	eukaryotic translation initiation
Cltb	clathrin	Epb4.1l2	erythrocyte protein
Commd6	COMM domain	Fam193b	family with sequence similarity193
Dclk2	doublecortin-like kinase	Hook2	hook homolog 2
Dedd2	death effector domain	Pcmtd2	protein O-methyltransferase
Dync1li2	dynein	Tpm3	tropomyosin 3,
Fam161a	family with sequence similarity	Arr3	arrestin 3, retinal
Fam184a	family with sequence similarity184	Gprasp2	G protein-coupled receptor
Ggps1	geranylgeranyl diphosphate synth	Gse1	genetic suppressor element
Gripap1	GRIP1 associated protein	Nsmaf	neutral sphingomyelinase
Impg2	interphotoreceptor matrix protein	Sgip1	SH3-domain GRB2-like
Plekha6	pleckstrin homology domain	Ubtf	upstream transcription factor
Ppie	peptidylprolyl isomerase E	Unc79	unc-79 homolog
Ptprf	protein tyrosine phosphatase	Ccdc64	coiled-coil domain containing
Samhd1	SAM domain and HD domain	Cugbp2	CUGBP, Elav-like
Scamp3	secretory carrier mem protein	Dctn6	dynactin 6
Smc5	structural maintenance of chrom	Mak	male germ cell-associated kinase
Sorbs1	sorbin and SH3 domain	Mare	nitrogen permease regulator-like 3
Trpm1	transient receptor cation channel	Scarb1	scavenger receptor class B,
Tsc2	tuberous sclerosis 2	Slc30a9	solute carrier family 30 (Zn)
Zcrb1	zinc finger CCHC-type	Trnau1ap	tRNA selenocysteine 1 protein
Zmiz2	zinc finger, MIZ-type	Whrn	whirlin Gene

It warrants mention that alternative splicing of transcripts is also prevalent in the retinas of normal animals. The 55 alternatively spliced transcripts therefore represent either novel spliced versions or spliced isoforms with altered abundance in diabetic animals, which suggests an association of alternative splicing with diabetic retinopathy. Noteworthy among these transcripts were those involved in neuronal cell survival, neural differentiation, apoptosis, endocytosis, intracellular transport, glucose metabolism and insulin impairment, G-protein coupled receptor signaling, and mRNA processing. Specifically, alternative splicing of arrestin (*Arr*), interphotoreceptor matrix proteoglycan (*Impg2*), and transient receptor membrane potential cation channel provide tentative evidence for the effects of diabetic retinopathy on the activity and integrity of photoreceptor cells in the retina.

### Responsiveness to treatments and molecular side effects of therapeutic intervention with candidate drugs

We have characterized the molecular responsiveness of animals to treatments as positive, negative, or as side effects, based on a comparison with wild type mice. We define *positive* effects of drugs as a reversal of transcript levels toward normalcy, and *negative* effects as changes in transcript levels of treated animals that are greater than those seen in the diabetic animals when compared to wild type animals. If a transcript remains unchanged in retinas from diabetic animals and the therapy leads to a significant change in its abundance, this is classified as a side effect.

Treatments with inhibitors of RAGE and p38 MAPK inhibited diabetes-induced changes (positive effects) and also exacerbated other changes (negative effects; Table 3, Table 4, Table 5, Table 6, Table 7, Table 8, Table 9, and Table 10). Both inhibitors blocked the diabetes-induced changes in levels of all crystallin transcripts and the levels of UDP-glucuronosyl transferases. Both inhibitors worked well in preventing the changes in the levels of Wnt pathway transcripts or apoptosis transcripts, but the RAGE-inhibitor appeared to work positively on a greater number of transcripts, as was the case for transcripts of inflammatory pathways. Surprisingly, photoreceptor transcripts were adversely affected in animals treated with either of these drugs. Of the 837 transcripts that were altered by greater than 1.5 fold in diabetic mice or in any one of the treatments, 379 transcripts were further exacerbated by RAGE inhibitor. The treatment with p38 MAPK inhibitor, on the other hand, caused further adverse exacerbations in 535 transcripts. Whether these exacerbations of transcript levels by drugs were beneficial or detrimental is not known at present.

**Figure 4:**
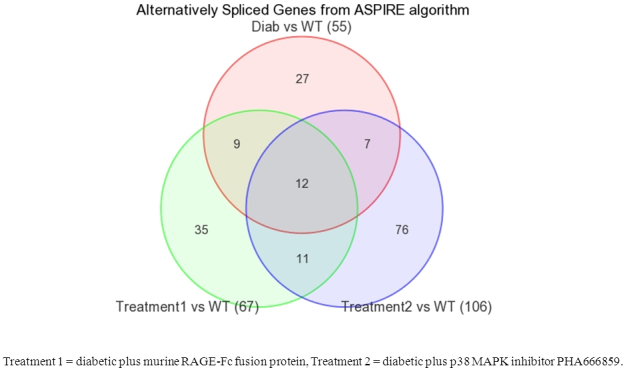
Unique and common splice forms of transcripts are present in various groups of animals. The splice variants from various samples were compared and the numbers of transcripts unique to a specific experimental pair or common to two or more experimental pairs are indicated.

Similar analyses were also performed with the data related to alternative splicing (Figure 4). Comparison of diabetic versus nondiabetic, RAGE inhibitor-treated diabetic versus nondiabetic and p38 MAPK inhibitor-treated diabetic versus nondiabetic animals led to the identification of alternatively spliced transcripts specific to each group of animals (Table 11, Table 12, and Appendix 2). Some notable alternatively spliced transcripts in diabetic animals included doublecortin-like kinase *Dclk3*, death effector domain *Dedd2*, dynein *Dync1li2*, interphotoreceptor matrix protein *Impg2*, ankyrin 2, arrestin 3, coiled-coil domain containing *Ccdc64*, G-protein coupled receptor *Gprasp2*, *Nsmaf*, dynactin 6, and whirlin (Table 11). Among the transcripts responsive to RAGE inhibitor were photoreceptor specific gene arrestin, *Nsmaf* transcript involved in apoptosis, and endocytosis transcript *Sgip1*. The exacerbated levels of 76 alternatively spliced transcripts in p38 MAPK inhibitor-treated animals included the Wnt pathway transcript *Axin1* and its target gene *Cldn1*, opsin transcript *Opnm1w*, *Mapk8ip3*, peroxisomal transcript *Pex16*, cilia transcript *Cep164*, and ocular regulator *Pax6* (Table 12 and Appendix 2). A comparison of alternatively spliced transcripts revealed 12 transcripts common among nondiabetic animals, diabetic animals, diabetic animals treated with RAGE inhibitor, and diabetic animals treated with p38 MAPK inhibitor (Figure 4). Among others, these genes included G-protein coupled receptor associated sorting protein, kinesin family member 1A, nuclear receptor co-repressor, phospholipase A2, and zinc finger protein 444. Although the summary presented in Figure 4 does not allow us to decipher the mechanisms of these therapies, the results suggest that the changes in splicing patterns in response to therapy may reflect the positions of p38 MAP kinase and RAGE in the cell signaling pathways relative to each other.

**Table 12:** Treatment responsive alternatively spliced transcripts.

Responsive to T1 and T2*		Unresponsive to T1 and T2*	Exacerbated by T2*			
Abca8a	Ggps1	Efha2	Ablim1	Epb4.1l3	Os9	Sag
Ankrd39	Gripap1	Fam169a	Adarb1	Fam177a	P4ha1	Sfmbt1
Apip	Impg2	Gprasp1	Asph	Fam57b	Papola	Slc16a3
Bdh1	Plekha6	Hnrnph3	Axin1	Fbxo3	Pax6	Srp54a
Cltb	Ppie	Kif1a	BC018101	Golga2	Pcdhga11	Srpk2
Commd6	Ptprf	Ncor1	BC037034	Golga4	Pcsk7	Srsf7
Dclk2	Samhd1	Pla2g5	Bod1l	Gpbp1	Pex16	Tacc2
Dedd2	Scamp3	RP23–285G5.1	Brsk2	Gpr85	Pfkp	Tlk2
Dync1li2	Smc5	Slc25a19	Cacna1a	Gtf2ird1	Pitpnm3	Trip12
Fam161a	Sorbs1	Strn	Cep164	Heg1	Pkp4	Ypel3
Fam184a	Trpm1	Zfp444	Cldnd1	Immt	Plekhb1	Zc3h11a
Zmiz2	Tsc2		Cnpy3	Ip6k2	Ppp2r3c	Zdhhc2
	Zcrb1		Col4a3bp	Iqsec3	Ppp6r2	Zfp207
			Crebl2	Itsn2	Pppde2	Zfp740
			Dlg4	Jmjd6	Ptcd3	Zfp788
			Dpp3	Mapk8ip3	Rab28	Zfp97
			Eno2	Mpp2	Ralgapa1	Zmynd8
			Nufip2	Msi1	Rufy3	Zscan29
			Opn1mw			
Responsive to T1*	Responsive to T2*	Exacerbated by T1 and T2*	Exacerbated by T1*			
Arr3	Agtpbp1	Ccdc64	Akap8	Dph3	Mthfs	Rho
Gprasp2	Ank2	Cugbp2	Amy2a2	Ehbp1	Nrcam	Sfrs14
Gse1	Dus1l	Dctn6	Amy2a3	Epb4.1	Pan3	Smarcc2
Nsmaf	Eif4g3	Mak	Amy2a4	Gm2382	Pdk1	Tmpo
Sgip1	Epb4.1l2	Mare	Ap1b1	Gm672	Phactr4	Unc45a
Ubtf	Fam193b	Scarb1	Arhgap12	Gmps	Ppfibp1	Zfp687
Unc79	Hook2	Slc30a9	Atrx	Ilf3	Prkcsh	Zmym3
	Pcmtd2	Trnau1ap	Cspp1	Maz	Prom1	
	Tpm3	Whrn	D4Ertd22e	Mettl3	R3hdm2	

*Treatment 1=diabetic plus murine RAGE-Fc fusion protein, Treatment 2=diabetic plus p38 MAPK inhibitor PHA666859.

## Discussion

This is the first report to describe the application of RNA-seq for comprehensive sequencing of transcripts in a diabetic retinopathy model and for evaluating the efficacy of candidate drugs. The RNA-seq methodology has allowed accurate and quantitative identification of molecular signatures. The quantitative RT–PCR data (by TaqMan or SYBR Green) validated the expression changes for all 15 transcripts that were examined. Interestingly, the rank order of differentially expressed transcripts (as a consequence of diabetes or treatment with drugs) differed between the two analysis methods used. The ELAND-SAM method used gene model annotations (RefGene) from UCSC, while the BWA-ANOVA method used the transcript model annotations (refflat) from UCSC. However, the transcripts revealed by both methods of analyses were verifiable by real-time PCR and thus the validity of both methods was supported. The validation of the RNA-seq platform for comprehensive transcriptome analysis has recently been described by Brooks et al. [24]. Given the growing use of next generation sequencing platforms, the development of additional analytical tools in the future should allow even better description of RNA-seq data.

As predicted, we observed changes in a variety of retinal transcripts due to diabetes. Some of these changes have been found to influence glucose metabolism or to alter the levels of hormone and hormone receptors in other tissues; these include glycerol-3-phosphate dehydrogenase, galanin receptor (*Galr2*) involved in G-protein coupled receptor signaling, transient receptor (*Trpm4*) involved in insulin secretion, insulin like growth factor binding proteins (*Igfbp3* and *Igfbp5*), the kinesin family of proteins, and calpain. Since vascular cells occupy such a small portion of the retina, the majority of observed changes likely are due to changes in the retinal neuroglia. Consistent with this idea, the list of transcripts that were significantly altered in diabetes did not contain transcripts corresponding to retinal vasculature, but targeted analyses of specific transcripts (such as endothelin-2, endothelin-3 and *VEGFB*) nevertheless revealed diabetes-induced alterations of 1.1–1.4 fold in levels of these transcripts.

Inflammation has been previously demonstrated to play an important role in the pathogenesis of diabetic retinopathy in animals [38,39]. Previous microarray profiling in a rat diabetic retinopathy model indicated changes in the levels of a variety of transcripts involved in inflammatory pathways [19]. Our RNA-seq data showed many inflammation-associated genes with altered expression; these included C1q TNF-related gene 5 (*C1qtnf5*), latent TGF-β binding protein (*Ltbp1*), phospholipase (*Pla2g2f*), interleukin 17 receptor 7c (*Il17rc*), and *C4b*. The changes in relatively few transcripts of the inflammatory pathway by eight months of diabetes seen in our study, when compared to changes reported at shorter durations, might indicate that inflammation is most severe early during the etiology of diabetic retinopathy, and that its severity might diminish as the disease progresses.

A variety of transcripts were altered in late stage diabetes; our RNA-seq data identified the Wnt signaling pathway, crystallins, and various transcription factors. Significant alterations were observed in the abundance of transcripts corresponding to *Wnt7b*, secreted frizzled receptor related protein-1 (*Sfrp1*), dapper antagonist of β-catenin (*Dact1*), Wnt inducible secreted protein-1 (*Wisp1*), and membrane frizzled related protein (*Mfrp*). Modulation of the Wnt pathway has been reported as a likely target for intervention for diabetic retinopathy [40], and *Wisp1* is known to upregulate the expression of the anti-apoptotic *Bcl-X(L)* gene [41]. In addition to the involvement of Wnt pathway proteins in other inflammatory diseases [42], alterations in Wnt signaling proteins have been shown in a rat diabetic retinopathy model [43].

We and others have previously observed a significant upregulation of different crystallin transcripts in diabetic retina [44]. The levels of fourteen crystallins increased significantly in diabetic mice. These proteins were initially characterized as structural components of the eye lens [45,46], but expression of crystallins has been shown in other tissues including the retina [47]. Crystallins that belong to the small heat shock protein family have a non-structural role [48], and retina-specific elevated levels of *Cryaa*, *Cryba1*, *Crygs*, *Crybb2*, *Crygb*, and *Crygc* have been reported in experimental uveitis [49]. *Cryaa* was shown to localize to photoreceptor cells in experimental uveitis, which likely represents a function in these cells to protect against apoptosis [49]. Our results clearly indicate a dramatic upregulation of various crystallins in diabetic retinopathy and suggest this to be a stress response, which may be similar to the reported effects of increased levels of *Hspb1* transcript in retina [50]. Post-translational modifications have been reported to alter chaperone activity of crystallins [51–53], while glycation associated with aggregation is likely to compromise the protective effects of crystallins in diabetic animals. Transcriptional upregulation of crystallins may therefore possibly represent a stress response, but the translated proteins may be rendered dysfunctional by diabetes-induced and deleterious post-translational modifications and other crosslinking events.

The deleterious changes to the retina that occur in diabetes are likely to be associated with alterations in the levels of transcripts associated with apoptosis, survival, and metabolic injury. Consistent with this hypothesis, we have observed changes in a variety of transcripts associated with apoptosis pathways. The metabolic injury to the neural retina is suggested by the altered levels of transcripts that modulate oxidative stress in cells. Changes in transcripts such as cytochrome c oxidase, glutathione-S-transferase omega-1 and omega-2, microsomal glutathione S-transferase, and glutathione synthase indicate that cellular injury to the retina occurs due to oxidative stress. While cytochrome oxidase is a sink for molecular oxygen channeled through electron transport chain in mitochondria, glutathione synthase and glutathione-S-transferases are important components of redox reactions.

The comparative analysis of gene level expression data also revealed differential expression of UDP-glucuronosyl transferases in diabetic animals. The UDP-glucuronosyl transferases metabolize a variety of xenobiotics, but they are also important for reactions occurring in cellular metabolism, such as those involving steroid hormones. We speculate that these enzymes may possess some other signaling activities, in line with the reported observation that UDP-glucuronosyl transferase 2B17 depletion led to downregulation of the *Mcl-1* gene [54]. If these types of signaling activities associated with UDP-glucuronosyl transferases are involved in the transcription of diabetic retinopathy-related genes, then targeted inhibition of specific UDP-glucuronosyl transferases might have therapeutic implications for diabetic retinopathy.

Numerous groups have been exploring ways to inhibit diabetic retinopathy by blocking specific metabolic pathways. Although none of these targeted approaches have been successful in the clinic to date, several candidate drugs are being evaluated for efficacy. The alterations in the levels of superoxide, nitric oxide, cyclooxygenase-2, *VEGF*, and *NF-κβ* in the early stages of diabetic retinopathy are consistent with the inflammation observed in retinal tissue. Our drug studies suggest that diabetes-induced alterations observed in inflammatory pathway transcripts are mediated in particular via p38 MAPK [40,55,56]. Another metabolic consequence of chronic hyperglycemia, in addition to inflammation, is the accumulation of advanced glycation end products (AGEs) [57]. These AGEs and other ligands are recognized by RAGE, and activated RAGE is associated with a variety of downstream consequences such as upregulation of *VEGF* and *NF-κβ*, and increased leucocyte adhesion in retinal microvascular endothelial cells, all of which are consistent with inflammation. Importantly, inhibitors of RAGE and p38 MAPK have been shown to inhibit the development of early stages of diabetic retinopathy in animals [3,25].

Since the phenotypic benefits of the two therapies are similar, assessing their efficacies against the molecular changes that they cause might provide important information. We have tested these effects to determine whether the drug prevents diabetes-induced changes in transcripts or further exacerbates diabetes-induced changes in transcript levels. Our data suggest that the RAGE inhibitor reversed the levels of a large number of transcripts whose levels were significantly altered in diabetic mice. Although the p38 MAPK inhibitor seemed comparably effective at inhibiting the development of diabetic retinopathy, it did not inhibit as many diabetes-induced changes in levels of transcripts, in contrast to what was seen with the RAGE inhibitor. One possible interpretation of this difference is that the transcripts normalized by inhibition of RAGE, but not p38 MAPK, are not critical for the development of the lesions that characterize early diabetic retinopathy. Interestingly, the p38 MAPK inhibitor caused further exacerbations in many more diabetes-induced changes in transcript levels than did the RAGE inhibitor. The intracellular protein p38 MAPK is downstream of RAGE [58] in one of the three pathways, such as PI3K, NF-kB, and MAPK that are initiated by AGE. Thus, one might expect a greater number of altered transcripts upon activation of RAGE, and reversal of transcript changes in animals treated with the inhibitor of RAGE.

An important and useful outcome of the RNA-seq approach was the identification of alternatively spliced transcripts during the development of diabetic retinopathy. Disease specific alternatively spliced transcript variants are shown to exist in various conditions [58,59], and our experimental studies show that alternatively spliced transcripts arise as diabetic retinopathy develops. Characterization of alternatively spliced transcripts that are specifically present or absent in a specific condition provides another opportunity for therapeutic intervention of diabetic retinopathy. Tocotrienol and EGCG are known to target splice sites and to modulate the abundance of alternatively spliced transcripts [60,61]. Exploration of the ability of these agents to inhibit diabetic retinopathy will be an interesting avenue of further research.

Reversal of the levels of alternatively spliced exons upon treatments also provides an additional validation of drug efficacy. Our novel finding of alternative splicing of transcripts in diabetes raises the possibility that these changes contribute to the pathogenesis of the retinopathy. From this perspective, the identification of photoreceptor-specific changes in arrestin, *Impg2*, and *Trpm1* raises the further possibility of a contribution by photoreceptors to diabetic retinopathy. These possibilities remain to be tested experimentally.

Just as therapies were shown above to promote further exacerbation of some diabetes-induced changes in transcript levels, therapy-specific exacerbation of alternative splicing also occurs. These side effects were more prominent following treatment with the p38 MAPK inhibitor than with the RAGE inhibitor, but the significance of these differences is not known at present. It warrants mention that alternatively spliced transcripts have been identified in a variety of diseases [62], and that a specific spliced form of a gene such as *COX-1* is one target of acetaminophen [59].

In conclusion, we have applied RNA-seq strategy and two analysis methods to determine the molecular signatures that involve representative transcripts of retinal vasculature, inflammatory pathway, Wnt signaling, apoptosis, crystallins, and UDP-glucuronosyl transferases. The importance of several of these abnormalities in the development of diabetic retinopathy has been identified previously, illustrating the power of the RNA-seq technique to identify important changes in disease. Of importance, however, is the identification of disease and treatment-specific alternative splicing by this strategy. The remarkable longevity of changes observed in transcript levels during the 8 months of diabetes in our study suggests that these changes may be necessary to promote and maintain the complications of diabetes. The inhibitors of RAGE and p38 MAPK reversed some of the diabetes-induced levels of various transcripts to normal levels, and exacerbated the changes in others. The significance and mechanisms underlying these alterations remain to be determined, but our results suggest RNA-seq as a desirable strategy for investigating the pathogenesis of diabetic retinopathy or other diseases, to distinguish between candidate drugs and to determine their mechanisms of action.

## Appendix 1. Comparison of transcript abundance changes obtained by two methods of analysis*.

To access the data, click or select the words “Appendix 1.” This will initiate the download of a compressed (pdf) archive that contains the file. *Method I (BWA-ANOVA) and method II (ELAND-SAM) have been described in the methods’ section. Fold changes are calculated as a ratio of transcripts in diabetic animals and nondiabetic controls.

## Appendix 2. Treatment responsive alternatively spliced transcripts.

To access the data, click or select the words “Appendix 2.” This will initiate the download of a compressed (pdf) archive that contains the file. *Treatment 1=diabetic plus murine RAGE-Fc fusion protein, Treatment 2=diabetic plus p38 MAPK inhibitor PHA666859.
